# Response of Soil Microbial Community Structure Mediated by Sulfur-Induced Resistance Against Kiwifruit Bacterial Canker

**DOI:** 10.3389/fmicb.2022.883463

**Published:** 2022-05-30

**Authors:** Sen Yang, Ran Shu, Xianhui Yin, Youhua Long, Jun Yuan

**Affiliations:** ^1^Engineering and Technology Research Center of Kiwifruit, Guizhou University, Guiyang, China; ^2^Institute of Crop Protection, Guizhou University, Guiyang, China

**Keywords:** sulfur, induced resistance, kiwifruit bacterial canker, soil microorganisms, soil microbial diversity

## Abstract

Kiwifruit bacterial canker caused by *Pseudomonas syringae* pv. *actinidiae* (Psa) is a major threat to kiwifruit worldwide, and effective control measures are still lacking. Sulfur, as a mineral, has been proved to improve plants' resistance to pathogens. It is of great significance to study the effect of sulfur on rhizosphere microorganisms in kiwifruit planting areas infected by Psa for controlling kiwifruit canker. In this study, the sulfur powder and organic fertilizer were mixed as base fertilizer to treat the soil in the area where kiwifruit bacterial canker occurs. We investigated the incidence of kiwifruit bacterial canker in 2018 and 2019 after sulfur application and the changes in microbial characteristics and community composition structure in the kiwifruit rhizosphere by using the plate-counting method and high-throughput sequencing technology. Fertilization treatments of kiwifruit roots with sulfur and organic fertilizer reduced kiwifruit bacterial canker severity. The diversity of soil microbial communities increased significantly after sulfur application in the range of 1.0~2.0 kg/m^3^. In particular, the bacterial genera level showed a high diversity after 2 years of sulfur application, reaching more than 516 genera. Furthermore, sulfur treatment resulted in a significant increase in specific microbial taxa, including members of the *Acidothe*rmus, *norank_f__HSB_OF53-F07*, and *norank_f __Acidobacteriaceae__Subgroup_1*. Moreover, the proportion of the dominant bacteria Acidothermus in the population showed an increasing trend. Altogether, the sulfur application is the key factor leading to microbial differences in kiwifruit rhizosphere soil. Appropriate sulfur can improve microbial structure characteristics of kiwifruit rhizosphere soil, increase bacterial diversity index, and change bacterial community composition structure.

## Introduction

Kiwifruit bacterial canker caused by *Pseudomonas syringae* pv. *actinidiae* (*Psa*) is an epidemic disease during the production of kiwifruit (Vanneste, 2017; Froud et al., 2018), which often results in sudden and severe economic losses and limitations in kiwifruit cultivation worldwide (Luti et al., 2021). People have explored various management strategies for controlling this disease (Bai et al., 2022), including but not limited to the application of antibiotics or copper and the breeding of resistant varieties (Wicaksono et al., 2018). However, available measures for this disease remain insufficient. In addition, these measures have certain limitations, such as phytotoxicity, induction of bacterial resistance, pesticide residues, or time consumption. Therefore, it is necessary to explore alternative strategies for controlling kiwifruit bacterial canker.

Soil microorganisms in natural ecosystems play a vital role in promoting crop growth and controlling plant diseases, which can directly or indirectly affect the composition and biomass of plant communities (Mendes et al., 2011). The diversity and uniformity of soil microbial community structure could affect the stability of the ecosystem and improve the crops' ability to respond to the changes in the soil microecological environment, especially the ability to resist pathogenic microorganisms and increase crop resistance to disease (Berendsen et al., 2012). Researchers have explored a variety of measures to improve plant resistance by changing the soil microbial population. For example, Karunakaran et al. (2013) found that different silicon sources added to soil had other effects on the abundance of soil bacterial communities and beneficial bacteria in the soil rhizosphere. Liu et al. (2019) confirmed that soil selenium decreased the sclerotinia stem rot (SSR) severity by increasing the microbiome diversity in soil (Liu et al., 2019). Mannaa et al. (2020) demonstrated that acibenzolar-s-methyl was effective at suppressing pine wilt disease, and several ecologically important microbial taxa were significantly altered after treatment with two resistance-inducing chemical elicitors. Therefore, investigation of the soil microbial community structure in the area where kiwifruit bacterial canker occurs is of benefit to developing effective bacterial canker management measures.

Sulfer is a suitable soil amendment, which can improve the soil's microecological environment and promote crops' nutrient absorption (Zakari et al., 2021). Our previous study found that sulfur significantly promoted the growth, development, and quality of kiwifruit and induced the resistance of *Actinidia chinensis* and *Actinidia deliciosa* to *Psa*. The control effect was more than 70% in greenhouse and field (Long et al., 2017; Gu et al., 2021). Zhang et al. (2021) and Gu et al. (2021) explored the sulfur-induced kiwifruit resistance to canker caused by *Pseudomonas syringae* pv. *actinidiae via* the salicylic acid signaling pathway and morphological structure modification in the kiwifruit stems. However, what effect does the sulfur application have on soil microbial community structure and diversity of kiwifruit? After sulfur application, what is the relationship between soil microbial community and kiwifruit resistance to disease? All these questions need to be confirmed by our further research. This study explores the impact of different sulfur contents on the rhizosphere soil microorganisms of kiwifruit by using the traditional plate number method and Illumina Miseq method. The research results will provide a basis for rational use of induced resistance to control kiwifruit canker disease.

## Materials and Methods

### Test Materials

The content of sulfur powder in the test was 95% (Hebei Shuangji Chemicals Company, Hebei, China). The total nutrient content in the refined organic fertilizer was > 4% and the organic matter content was ≥30% (w/v) (Guizhou Jilong Ecological Science and Technology Co., Guiyang, China). The kiwifruit variety used was “Guichang,” and the vines were 17 years old.

### Experimental Site

**Figure 1:**
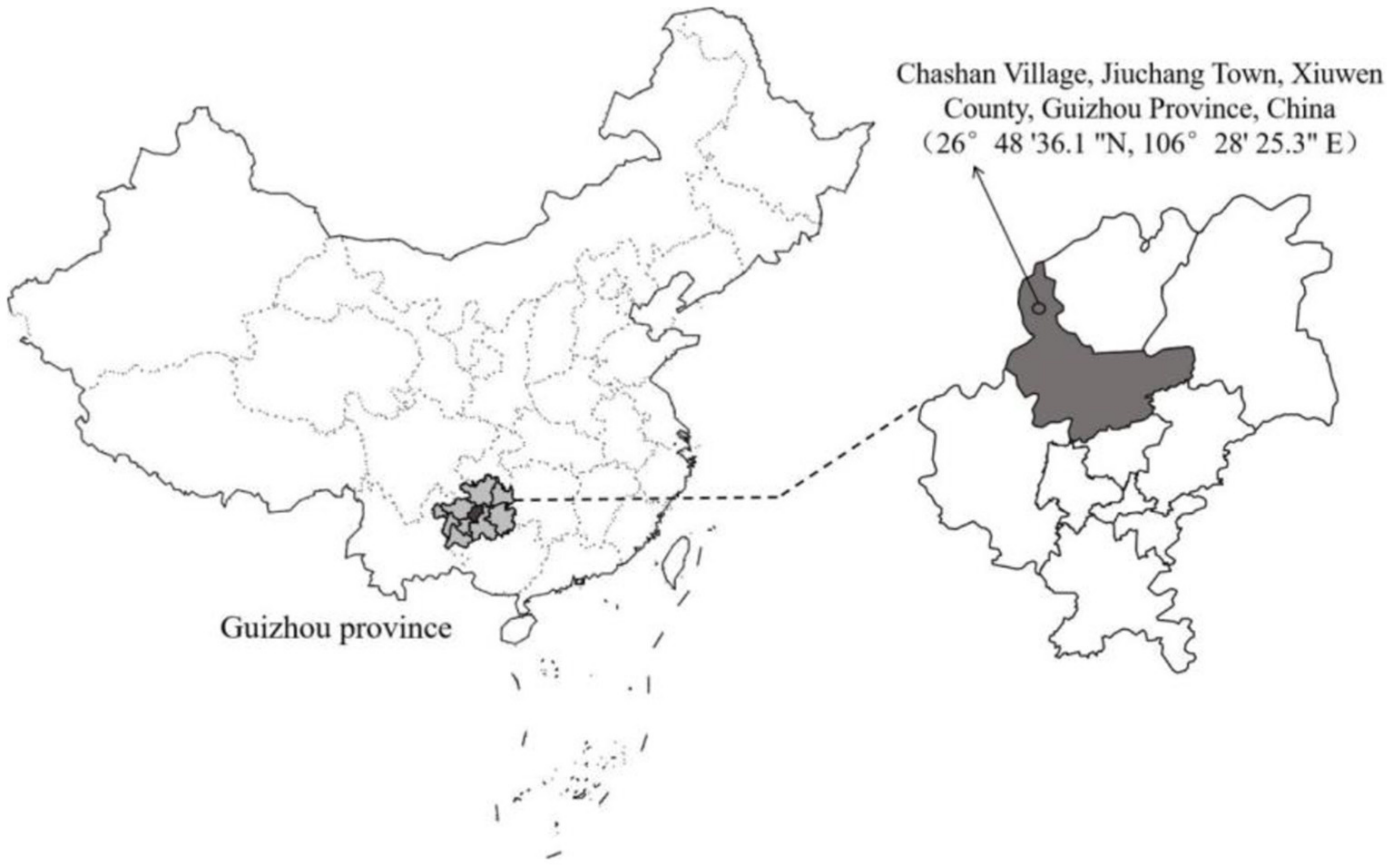
The map of the experimental site.

On January 17, 2017, the experiment was carried out in the kiwifruit planting base (26°48′ 36.1″ N, 106°28′ 25.3″ E, above sea level 1,321 m) in Chashan Village, Jiuchang Town, Xiuwen County, Guizhou Province, China (Figure 1). The site is a subtropical monsoon humid climate with an average temperature of 16°C and an annual rainfall of 1,293 mm. The soil type of the orchard was yellow soil. All the kiwifruit vines were planted north-south with a planting density of 3 m × 4 m (plant distance × row spacing). A total of 1,110 kiwifruit vines were planted per hectare, among which 68 were female, and the trees grew uniformly. Before the experiment, the soil background value was measured in the orchard by random multi-point mixed soil. The pH, organic matter content, total nitrogen content, alkali-hydrolyzed nitrogen content, and available phosphorus content were 7.64, 30.17 g/kg, 2.02 g/kg, 109.43 mg/kg, and 4.63 mg/kg, respectively. The available potassium content was 106.67 mg/kg and available sulfur was 13.05 mg/kg. Kiwifruit canker disease seriously occurred in the orchard and the incidence rate reached 76.8% in 2016.

### Experimental Design and Sampling

According to the kiwifruit crown size estimation, each vine's root-soil volume was calculated as 1 to 1.5 m^3^. The field experiment consisted of seven sulfur concentration treatments (S_0_, S_0.5_, S_1.0_, S_1.5_, S_2.0_, S_2.5_, and S_3.0_). Twenty-eight test plots were set with 10 kiwifruit plants in each plot and randomly divided into four groups using a single factor randomized block design. Every treatment group consisted of soil, 10 kg organic fertilizer, and a different concentration of a sulfur powder. The sulfur contents were 0, 0.5, 1.0, 1.5, 2.0, 2.5, and 3.0 kg/m^3^ and the sulfur powder was added to 1 m^3^ soil. The treatment containing soil, 10 kg organic fertilizer, and 0 kg/m^3^ sulfur powder was used as the control (S_0_). After sulfur powder and organic fertilizer were mixed evenly, each vine was applied with the annular ditch method (Gu et al., 2021) as base fertilizer. Protection lines were set up between each plot. All treatments were managed in the same field according to the local kiwifruit cultivation techniques.

### Investigation on the Incidence of Kiwifruit Canker Disease

After applying sulfur in the orchard, the incidence of canker disease was investigated in the bleeding period of kiwifruit (April) for 2 consecutive years. The incidence rate was calculated in the following way:

Incidence rate (%) = (The number of plants showing disease symptoms / the total number of investigated plants) × 100

### Determination of the Microbial Quantity and Characteristics

After applying sulfur for 2 years, the rhizosphere soil near the root of kiwifruit 20 cm from the surface was collected and put into a sterilized plastic bag, placed in a moving 4°C thermostatic chamber, and brought back to the laboratory to determine the number of microorganisms and their characteristics. The soil microorganism was separated by the plate dilution method. The fungi were isolated using the Madin Rose Bengal Red-Chloramphenicol Agar medium. Nutrient agar (Na) medium was used to isolate soil bacteria and Gao's 1 medium was used to isolate actinomycetes. In addition, soil microbial biomass carbon (MBC) was determined by the chloroform fumigation-K_2_SO_4_ extraction method and soil microbial biomass nitrogen (MBN) was determined by the chloroform fumigation-K_2_SO_4_ extraction-flow injection nitrogen analyzer method (Xiao et al., 2019).

### DNA Extraction, Library Preparation, and Sequencing

Soil samples of 0~20 cm rhizosphere of kiwifruit were collected after 1 and 2 years of sulfur application. The soil samples were put into 50 mL centrifuge tubes, sealed and wrapped with tin foil, and put into−80°C liquid nitrogen and brought back to the laboratory.

From 1 g of soil, total DNA from the soil samples was extracted using the E.Z.N.A.^®^ SOIL kit (Omega Bio-Tek, Norcross, GA, USA), and DNA concentration and purity were determined using NanoDrop 2000. After testing DNA quality, the MiSeq sequencing platform provided by Shanghai Meji Biological Company was adopted. PCR amplification was performed using the V4 region primers of bacterial 16S rDNA (338F and 806R), the ITS1 region primers of fungal ITS (ITS5-1737F and ITS2-2043R), and the V3 region primers of actinomycete 16S rRNA (341F and 518R). In addition, the amplification procedure was as follows: pre-denaturation at 95°C for 3 min was followed by 27 cycles (denaturation at 95°C for 30 s, annealing at 55°C for 30 s, and extension at 72°C for 30 s) and final extension at 72°C for 10 min (PCR instrument: ABI Geneamp^®^ 9700). The total amplification system was 20 μl, including 4 μl 5×FastPFU buffer, 2 μl 2.5 mM dNTPs, 0.8 ul primer (5 uM), 0.4 ulFastPFU polymerases, and 10 ng DNA template. PCR products were recovered using 2% agarose gel and purified using AxyPrep DNA Gel Extraction Kit (AxyGenBiosciences, Union City, CA, USA). Then, Tris-HCl elution and 2% agarose electrophoresis were performed. Quantification was performed using the Quantifluor™-ST (Promega, USA).

### Data Preprocessing

The data obtained by Miseq sequencing are double-ended sequence data. First, pairs of reads were merged into a sequence according to the overlapping relationship between P.E. reads, and then the quality of reads and the effect of the merge were controlled and filtered simultaneously. According to the barcode and primer sequences at both ends of the sequence, the samples are distinguished to get the effective sequence. Then, the direction of the sequence is corrected, which is the optimization data. Using UPARSE software (version 7.1), http://drive5.com/uparse/, according to the similarity of 97% to OTU sequence clustering, we got rid of a single sequence in the process of clustering and chimeras. Species classification was annotated for each sequence using RDP Classifier (http://rdp.cme.msu.edu/) compared with the Silva database, and the comparison threshold was set at 70%.

### Statistical Analysis

Data were expressed as means ± standard deviation (Sd), and statistically significant differences between treatments were tested using one-way ANOVA. All statistical tests were conducted using SPSS 18.0 software (SPSS Inc., Chicago, IL, USA), and the means were separated using the least significant difference (LSD) test at *p* < 0.05. Vegan package and PCA package in R language were used for a heatmap and principal component analysis (PCA) of species community richness in rhizosphere soil microorganisms (bacteria, actinomycetes, and fungi).

## Results

### Incidence of Kiwifruit Canker After Different Sulfur Treatments

**Figure 2:**
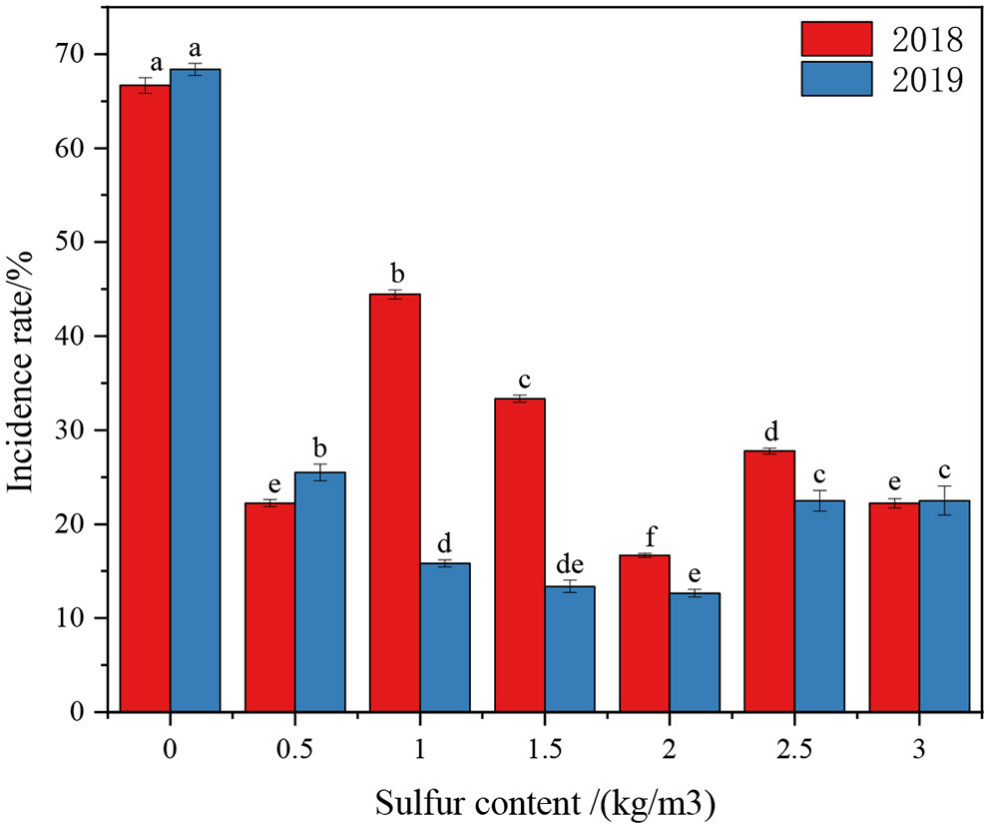
Effects of different sulfur treatments on the incidence rate of kiwifruit canker. Different letters indicate statistically significant differences (*p* < 0.05) between groups in each column. The same as below.

In 2018 (1 year after sulfur application), the results we obtained showed that the incidence of kiwifruit canker was higher in the control group, reaching 66.67% (Figure 2). Compared with the control group, different concentrations of sulfur treatment can significantly reduce the incidence of kiwifruit canker (<45%), indicating that sulfur application had a certain control effect on kiwifruit canker. The lowest incidence rate was 16.67% when the sulfur application rate was 2.0 kg·m^−3^. In 2019 (after 2 years of sulfur application), kiwifruit canker occurred seriously in the control group, with an incidence of 68.38%. To sum up, the incidence of kiwifruit canker after the sulfur application was further decreased compared with that in 2018. The lowest incidence rate was only 12.26% at the sulfur concentration of 2.0 kg·m^−3^. The results indicated that sulfur application could improve the disease resistance of kiwifruit, thus reducing the incidence of kiwifruit canker.

### Effect of Sulfur on Microbial Quantity and Characteristics in Rhizosphere Soil of Kiwifruit

As shown in Table 1, the microorganism quantity in the rhizosphere soil of kiwifruit increased after 2 years of sulfur application. When the vines were treated with 1.0 kg/m^3^, the number of bacteria was 94.00 × 10^5^ CFU/g, which is significantly different from the control. In comparison, when sulfur was applied at 1.5 kg/m^3^, the number of bacteria was 60.67 × 10^5^ CFU/g, which was 43.61 and 12.64% higher than the control (S_0_), respectively. Thus, the difference between the two treatment groups was significant. A low concentration of sulfur treatment could promote the growth of fungus numbers, while a high concentration of sulfur treatment can not. The number of fungi in the rhizosphere soil of kiwifruit was significantly increased by 89.00% in the treatment of 0.5 kg/m^3^. The fungi began to be suppressed when the sulfur concentration increased to 2.5 and 3.0 kg/m^3^. When the sulfur application content was 2.5 kg/m^3^, the number of actinomycetes was the highest, which was 1.55 × 10^4^ CFU /g, and the difference was significant compared with the control.

**Table 1:** Effects of sulfur on the microbial characteristics of the kiwifruit rhizosphere soil.

**Treatment**	**Bacteria (× 10^**5**^ cfu/g)**	**Pi (%)**	**Fungi (× 10^**4**^ cfu/g)**	**Pi (%)**	**Actinomyces (× 10^**4**^cfu/g)**	**Pi(%)**	**Total amount of microorganisms (× 10^**5**^ cfu/g)**	**SMBC (mg/kg)**	**SMBN (mg/kg)**	**DI**
S_0_	53.00 ± 4.6^b^	99.87	0.33 ± 0.58^b^	0.06	0.33 ± 0.23^c^	0.06	53.07	395.33 ± 15.17^c,d^	38.37 ± 1.40^c^	0.0105
S_0.5_	57.66 ± 7.26^b^	97.94	3.00 ± 1.73^a^	0.51	9.00 ± 1.41^b^	1.53	58.87	405.33 ± 2.51^b,c^	39.33 ± 1.01^b,c^	0.1112
S_1.0_	94.00 ± 20.77^a^	99.47	1.00 ± 0.57^b^	0.11	0.67 ± 0.47^c^	0.07	94.50	421.00 ± 9.16^ab^,	41.32 ± 0.99^a,b^	0.0177
S_1.5_	60.67 ± 5.10^b^	99.62	1.00 ± 0.58^b^	0.16	1.33 ± 0.94^c^	0.22	60.90	432.00 ± 8.892^a^	42.20 ± 0.71^a^	0.0277
S_2.0_	48.67 ± 4.09^b^	99.73	1.33 ± 0.33^b^	0.27	0.00 ± 0.00^c^	0.00	48.80	421.33 ± 3.21^a,b^	39.13 ± 2.15^b,c^	^§^–
S_2.5_	46.33 ± 8.29^b^	96.72	0.33 ± 0.33^b^	0.07	15.33 ± 3.29^a^	3.20	47.90	398.33 ± 8.33^c,d^	35.87 ± 1.11^d^	0.1474
S_3.0_	47.67 ± 8.05^b^	99.23	0.33 ± 0.33^b^	0.07	0.33 ± 0.23^c^	0.07	48.04	382.67 ± 8.51^d^	31.78 ± 1.43^e^	0.0177

§ *–: indicating that one or more microorganisms are not isolated. Pi: indicates the percentage of the total species of microorganisms. SMBC, Soil microbial biomass C; SMBN, Soil microbial biomass. All data were obtained from a representative assay, the mean of four identical replicates ± Sd*.

*Different lowercase letters represented a significant difference (P < 0.05)*.

When the sulfur application content was between 0.5 kg/m^3^ and 1.5 kg/m^3^, the number of microorganisms was in the state of excitation, and the total amount of microorganisms ranged from 58.87 × 10^5^ CFU/g to 94.50 × 10^5^ CFU/g, which could increase the total amount of microorganisms in the root-soil of kiwifruit (Table 1). However, when the sulfur application rate was between 2.0 kg/m^3^ and 3.0 kg/m^3^, the total microbial biomass was inhibited. The total microbial biomass of each treatment was less than that of the control without sulfur application.

The microbial diversity index (DI) of all treatments except 2.0 kg/m^3^ was higher than that of S_0_ control, and the diversity index of S_2.5_ was the highest (0.1474), which was 92.88% higher than that of S_0_ control. When the sulfur application content was between 0.5 and 3.0 kg/m^3^, all treatments' microbial biomass carbon and nitrogen contents were higher than those of the no-sulfur treatment. However, the soil's microbial biomass carbon and nitrogen increased first and then decreased. The highest value of both treatments was found when the sulfur application rate was 1.5 kg/m^3^. Compared with the control group, the contents were 432.00 mg/kg and 42.20 mg/kg, respectively, which were increased by 8.49 and 9.08%, and the difference was significant. Therefore, the sulfur application between 0.5 kg/m^3^ and 2.0 kg/m^3^ could effectively increase the number of bacteria, fungi, and actinomycetes in the root-soil of kiwifruit and increase the total and effectively improve total carbon and nitrogen content of soil rhizosphere microbial biomass. The results indicated that proper sulfur application could effectively improve the microbial diversity of kiwifruit root-soil.

### Effect of Sulfur on α Diversity of Kiwifruit Rhizosphere Soil Microorganisms

#### Bacteria

After 1 year of sulfur application in kiwi soil, 27 phyla, 67 classes, 127 orders, 231 families, 405 genera, and 711 species were detected by the high-throughput sequencing. A total of 33 phyla, 76 classes, 156 orders, 288 families, 516 genera, and 952 species were seen after 2 years of sulfur application. As shown in **Table 3**, at the 97% classification level, the average coverage rate of all samples was above 99%. After 1 year of sulfur application, the Shannon index of each sulfur application treatment was smaller than that of no sulfur application treatment. However, the indexes of SOBS, ACE, Chao, and Simpson in S_2.5_ treatment were higher than those in no sulfur treatment, and the Chao index in S_2.5_ treatment was significantly higher than that in no sulfur treatment (*P* < 0.05). After applying sulfur for 2 years, the indexes of Shannon, ACE, and Chao increased with the increase of sulfur concentration, but the Simpson index was contrary. In general, sulfur treatment can improve the bacterial diversity of kiwifruit rhizosphere soil (Table 2).

**Table 2:** Sequence statistics and diversity indexes of sulfur on kiwifruit rhizosphere soil bacteria.

	**Treatment**	**Taxonomic level in 0.97**					
		**Sobs**	**Shannon**	**Simpson**	**ACE**	**Chao**	**Coverage**
Sulfur application for 1 year	S_0_	1214.00 ± 52.00	6.09 ± 0.16^a^	0.0051 ± 0.0005^d^	1315.57 ± 30.33^a,b^	1308.51 ± 9.32^e^	0.9929 ± 0.0043
	S_0.5_	1194.00 ± 12.00	5.70 ± 0.07^a,b, c^	0.0124 ± 0.0005^b^	1348.42 ± 4.16^a^	1368.85 ± 5.42^b^	0.9905 ± 0.0015
	S_1.0_	1170.00 ± 19.00	5.89 ± 0.10^a,b^	0.0071 ± 0.0015^d^	1306.15 ± 4.44^b^	1323.38 ± 3.16^d,e^	0.9915 ± 0.0011
	S_1.5_	1212.00 ± 13.00	5.83 ± 0.06^a,b^	0.0081 ± 0.0007^c,d^	1336.72 ± 1.38^a,b^	1357.41 ± 1.55^b,c^	0.9915 ± 0.0008
	S_2.0_	1188.00 ± 8.00	5.34 ± 0.17^c^	0.0269 ± 0.0015^a^	1317.03 ± 6.81^a,b^	1325.57 ± 2.96^d^	0.9913 ± 0.0018
	S_2.5_	1244.00 ± 18.00	5.84 ± 0.07^a,b^	0.0106 ± 0.0011^b,c^	1355.15 ± 4.89^a^	1386.76 ± 4.57^a^	0.9917 ± 0.0029
	S_3.0_	1185.00 ± 22.00	5.57 ± 0.16^b,c^	0.0131 ± 0.0005^b^	1344.57 ± 2.80^a,b^	1347.56 ± 4.27^c^	0.9904 ± 0.0057
Sulfur application for 2 years	S_0_	1243.50 ± 105.02	5.55 ± 0.28	0.0180 ± 0.0065^a,b^	1519.93 ± 117.23	1544.13 ± 107.27	0.9799 ± 0.0015^a,b^
	S_0.5_	1216.00 ± 74.27	5.22 ± 0.36	0.0398 ± 0.0207^a^	1549.28 ± 75.72	1585.47 ± 62.85	0.9780 ± 0.0011^b^
	S_1.0_	1178.75 ± 93.85	5.77 ± 0.24	0.0084 ± 0.0023^b^	1443.80 ± 81.01	1434.62 ± 91.47	0.9815 ± 0.0004^a,b^
	S_1.5_	1143.50 ± 93.69	5.40 ± 0.29	0.0216 ± 0.0069^a,b^	1446.31 ± 82.78	1439.07 ± 83.81	0.9805 ± 0.0013^a,b^
	S_2.0_	1239.00 ± 106.63	5.60 ± 0.27	0.0148 ± 0.0065^a,b^	1538.46 ± 126.08	1549.11 ± 100.81	0.9792 ± 0.0016^a,b^
	S_2.5_	1156.75 ± 25.11	5.66 ± 0.08	0.0123 ± 0.0027^a,b^	1398.17 ± 29.50	1373.59 ± 23.97	0.9824 ± 0.0006^a^
	S_3.0_	110.75 ± 1.03	3.72 ± 0.04	0.0483 ± 0.0036	159.85 ± 9.28	152.26 ± 5.76	0.9523 ± 0.0024

*Different lowercase letters represented a significant difference (P < 0.05)*.

#### Actinomycetes

After 1 year of sulfur application, a total of 1 phylum, 1 class, 14 orders, 33 families, 61 genera, and 94 species of actinomyces were detected. After 2 years, a total of 1 phylum, 1 class, 16 orders, 49 families, 86 genera, and 138 Species were seen. The indexes of Shannon, ACE, and Chao showed a decreasing trend with the increase of sulfur concentration after 1 year of sulfur application, while Simpson showed the opposite direction. There was no significant change in the SOBS index among all treatments, which fluctuated between 129.00 and 140.00 with no significant difference (*P* < 0.05). After 2 years, SOBS and Shannon indexes showed a downward trend with the increase in sulfur application concentration, but there was no significant difference between treatments (*P* < 0.05). Similarly, Simpson, ACE, and Chao indexes did not reach a considerable level among all treatments, indicating that sulfur treatment had no significant effect on soil actinomycetes diversity (Table 3).

**Table 3:** Sequence statistics and diversity indexes of sulfur on kiwifruit soil actinomycetes.

	**Treatment**	**Taxonomic level in 0.97**					
		**Sobs**	**Shannon**	**Simpson**	**ACE**	**Chao**	**Coverage**
Sulfur application for 1 year	S_0_	136.00 ± 5.13	4.25 ± 0.21^a^	0.0232 ± 0.0015^b^	139.89 ± 4.69	140.09 ± 2.15	0.9967 ± 0.0009
	S_0.5_	136.00 ± 5.13	4.08 ± 0.40^a^	0.0274 ± 0.0012^b^	141.43 ± 1.02	139.67 ± 6.12	0.9960 ± 0.0015
	S_1.0_	140.00 ± 6.02	4.33 ± 0.08^a^	0.0200 ± 0.0026^b^	145.51 ± 1.63	148.25 ± 1.51	0.9960 ± 0.0004
	S_1.5_	138.00 ± 4.35	4.21 ± 0.08^a^	0.0221 ± 0.0055^b^	146.31 ± 2.06	150.00 ± 2.07	0.9947 ± 0.0017
	S_2.0_	134.00 ± 3.05	3.59 ± 0.25^a,b^	0.0855 ± 0.0046^a^	141.28 ± 6.37	142.75 ± 1.46	0.9950 ± 0.0018
	S_2.5_	137.00 ± 2.64	4.14 ± 0.15^a^	0.0246 ± 0.0039^b^	142.24 ± 2.75	143.50 ± 4.17	0.9957 ± 0.0009
	S_3.0_	129.00 ± 4.04	3.23 ± 0.29^b^	0.0982 ± 0.0088^a^	144.50 ± 2.88	150.43 ± 1.51	0.9917 ± 0.0051
Sulfur application for 2 years	S_0_	132.50 ± 7.26	4.12 ± 0.12	0.0306 ± 0.0044	165.40 ± 9.76	154.34 ± 9.69	0.9553 ± 0.0042
	S_0.5_	123.25 ± 6.42	3.98 ± 0.15	0.0376 ± 0.0092	162.17 ± 7.72	161.71 ± 6.95	0.9523 ± 0.0029
	S_1.0_	114.25 ± 10.93	3.87 ± 0.29	0.0431 ± 0.0184	154.95 ± 4.24	142.68 ± 6.33	0.9592 ± 0.0028
	S_1.5_	113.75 ± 7.19	3.78 ± 0.16	0.0491 ± 0.012	162.59 ± 5.67	160.81 ± 8.70	0.9523 ± 0.0019
	S_2.0_	124.00 ± 6.01	4.05 ± 0.09	0.0329 ± 0.0052	170.48 ± 21.55	153.30 ± 11.50	0.9541 ± 0.0057
	S_2.5_	111.00 ± 3.11	3.75 ± 0.09	0.0513 ± 0.012	152.84 ± 12.31	145.51 ± 13.40	0.9568 ± 0.0034
	S_3.0_	110.75 ± 1.03	3.72 ± 0.04	0.0483 ± 0.0036	159.85 ± 9.28	152.26 ± 5.76	0.9523 ± 0.0024

*Different lowercase letters represented a significant difference (P < 0.05)*.

#### Fungi

As can be seen from Table 4, 7 phyla, 25 classes, 67 orders, 126 families, 236 genera, and 349 species of fungi were detected after sulfur application for 1 year. Eight phyla, 28 classes, 81 orders, 167 families, 334 genera, and 527 species were seen after 2 years of sulfur application. SOBS, ACE, and Chao indexes of the S_1.5_ treatment were the highest after 1 year, and the difference was significant compared with that of the S_0_ treatment (*P* < 0.05). Shannon's index of S_3.0_ was the highest, but the difference was not noticeable compared with the S_0_ treatment. Simpson index showed a decreasing trend with the increase of sulfur concentration. However, after sulfur application for 2 years, there was no significant difference in the fungal diversity index among all treatments, indicating that sulfur application had no significant effect on rhizosphere soil fungal diversity over time.

**Table 4:** Sequence statistics and diversity indexes of sulfur on kiwifruit rhizosphere soil fungi.

	**Treatment**	**Taxonomic level in 0.97**					
		**Sobs**	**Shannon**	**Simpson**	**ACE**	**Chao**	**Coverage**
Sulfur application for one year	S_0_	474 ± 4^b^	3.71 ± 0.05^a^	0.0583 ± 0.0018^d,e^	535.40 ± 1.42^b^	519.57 ± 0.61^b^	0.9973 ± 0.0003
	S_0.5_	221 ± 7^f^	1.66 ± 0.04^d^	0.2981 ± 0.0396^a^	456.57 ± 2.83^e^	393.44 ± 1.96^f^	0.997 ± 0.0008
	S_1.0_	305 ± 7e	2.86 ± 0.02^c^	0.1414 ± 0.0057^c^	372.91 ± 2.55^g^	374.38 ± 0.86^g^	0.9977 ± 0.0005
	S_1.5_	531 ± 6^a^	3.46 ± 0.12^b^	0.1076 ± 0.0044c^d^	607.56 ± 4.73^a^	585.56 ± 4.31^a^	0.9968 ± 0.0006
	S_2.0_	435 ± 5^c^	3.33 ± 0.13^b^	0.0847 ± 0.0029^d,e^	514.75 ± 2.15^c^	506.74 ± 2.38^c^	0.9969 ± 0.0008
	S_2.5_	357 ± 5^d^	2.70 ± 0.05^c^	0.2028 ± 0.0116^b^	427.24 ± 0.^f^	419.25 ± 2.60^e^	0.9974 ± 0.0016
	S_3.0_	429 ± 5^c^	3.74 ± 0.03^a^	0.0520 ± 0.0015^e^	477.14 ± 0.^d^	473.28 ± 3.83^d^	0.9977 ± 0.0008
Sulfur application for two years	S_0_	422 ± 33	2.84 ± 0.33	0.1786 ± 0.0675	587.34 ± 49.76	560.04 ± 48.84	0.9956 ± 0.0004
	S_0.5_	351 ± 24	2.54 ± 0.08	0.1909 ± 0.0272	598.84 ± 40.08	515.87 ± 19.01	0.9958 ± 0.0002
	S_1.0_	403 ± 39	2.78 ± 0.35	0.1816 ± 0.0783	574.19 ± 8.90	553.89 ± 30.57	0.9957 ± 0.0002
	S_1.5_	359 ± 80	2.56 ± 0.43	0.2299 ± 0.0647	496.10 ± 72.16	469.73 ± 85.42	0.9964 ± 0.0007
	S_2.0_	419 ± 56	2.76 ± 0.27	0.1859 ± 0.0592	601.60 ± 73.65	587.82 ± 63.71	0.9954 ± 0.0005
	S_2.5_	373 ± 30	2.88 ± 0.17	0.1337 ± 0.0261	584.06 ± 57.70	544.63 ± 42.13	0.9957 ± 0.0003
	S_3.0_	400 ± 23	3.01 ± 0.21	0.1447 ± 0.0254	527.82 ± 21.44	498.87 ± 23.36	0.9961 ± 0.0002

*Different lowercase letters represented a significant difference (P < 0.05)*.

### Changes in the Soil Microbial Community Structure in Kiwifruit Rhizosphere After Sulfur Application

#### Horizontal Relative Abundance of Microorganisms

**Figure 3:**
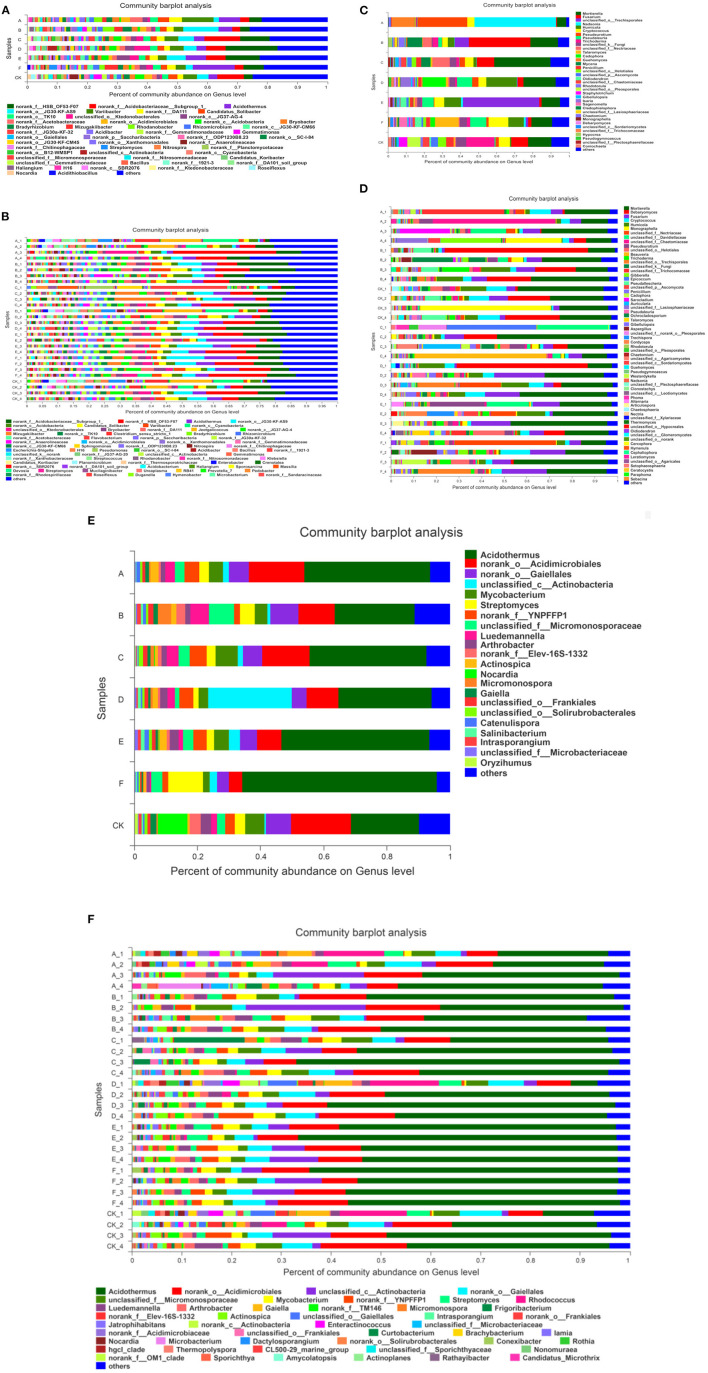
The horizontal abundance of microorganisms in kiwifruit soil after sulfur treatments. **(A,B)** bacteria; **(C,D)** fungi; **(E,F)** actinomycetes. A: 0.5 kg/m^3^ sulfur powder +10 kg organic fertilizer; B: 1 kg/m^3^ sulfur powder + 10 kg organic fertilizer; C: 1.5 kg/m^3^ sulfur powder + 10 kg organic fertilizer; D: 2.0 kg/m^3^ sulfur powder + 10 kg organic fertilizer; E: 2.5 kg/m^3^ sulfur powder + 10 kg organic fertilizer; F: 3.0 kg/m^3^ sulfur powder + 10 kg organic fertilizer; CK: only 10 kg organic fertilizer. The same below.

After 1 year and 2 years of sulfur application, 28 rhizosphere soil samples of kiwifruit orchard were sequenced, respectively, and the diversity of each sample was high at the genus level. As shown in Figure 3A, more than 405 genera of bacteria were detected after sulfur application for 1 year, among which are unknown bacteria and bacteria accounted for about 20% of the total sequencing. Among the known bacteria, *Acidithiobacillus norank_f__HSB_OF53-F07, norank_f __Acidobacteriaceae__Subgroup_1, Acidothermus, _norank-o-JG30-KF-AS9*, and *Bradyrhizobium* accounted for 18.42% to 20.57% of the total sequencing. With the increase of sulfur content, the abundance of dominant bacteria *norank_f__HSB_OF53-F07* first increased and then decreased. The abundance of *norank_f__HSB_OF53-F07* was the most obvious in S_2.0_, accounting for 17.26% of the whole sequence and 497.23% higher than S_0_. After applying sulfur for 2 years, all treatments also had a high diversity at the level of bacterial genera (Figure 3B), reaching more than 516 genera, among which the unknown bacteria accounted for about 11.55% to 29.74% of the total sequencing. The known bacteria include *Acidithiobacillus, norank_c__Cyanobacteria, Clostridium_sensu_stricto, norank_f__Acidobacteriaceae__Subgroup_1, norank_f__HSB_ OF53-F07*, and *Acidothermus*. Compared with no sulfur treatment, the proportion of *Acidothermus* in the population increased when the sulfur content increased.

More than 61 genera of actinomycetes were detected after 1 year of sulfur application (Figure 3C). The dominant species *Acidothermus* and *norank_o__Acidimicrobiales* accounted for 39.64% to 65.98% of the whole sequence. With the increase of sulfur content, the abundance of *Acidothermus* increased. The S_3.0_ treatment reached the maximum value of 61.72%, which increased by 185.21% compared with that of S_0_. The results indicated that sulfur application had a better effect on the abundance of Acidothermus. More than 86 genera of actinomycetes were detected after 2 years of sulfur application, among which Acidothermus was the dominant strain in all treatments, accounting for 5.43% to 64.86% of the total sequencing. The abundance of S_1.5_ kg/m^3^ was the highest in the sulfur application. It can be seen that sulfur application mainly increased the abundance of Acidothermus in the soil of kiwifruit (Figure 3D).

As shown in Figure 3E, fungi showed a high diversity at the genus level after sulfur application for 1 year, reaching more than 236 genera. The dominant fungi were *Mortierella, Fusarium*, and *unclassified_k__Fungi*, accounting for 6.17 to 53.54% of the whole sequence. The proportion of *Mortierella* in S_1.5_ significantly increased in the fungal population, which was 31.11%, and 140.78% higher than that in S_0_. After 2 years of sulfur application, the fungal flora reached more than 334, with *Mortierella, Fusarium*, and *Debaryomyces* as the main flora, accounting for about 20% to 40% of the total sequence (Figure 3F).

#### Main Components of the Microbial Community Structure

**Figure 4:**
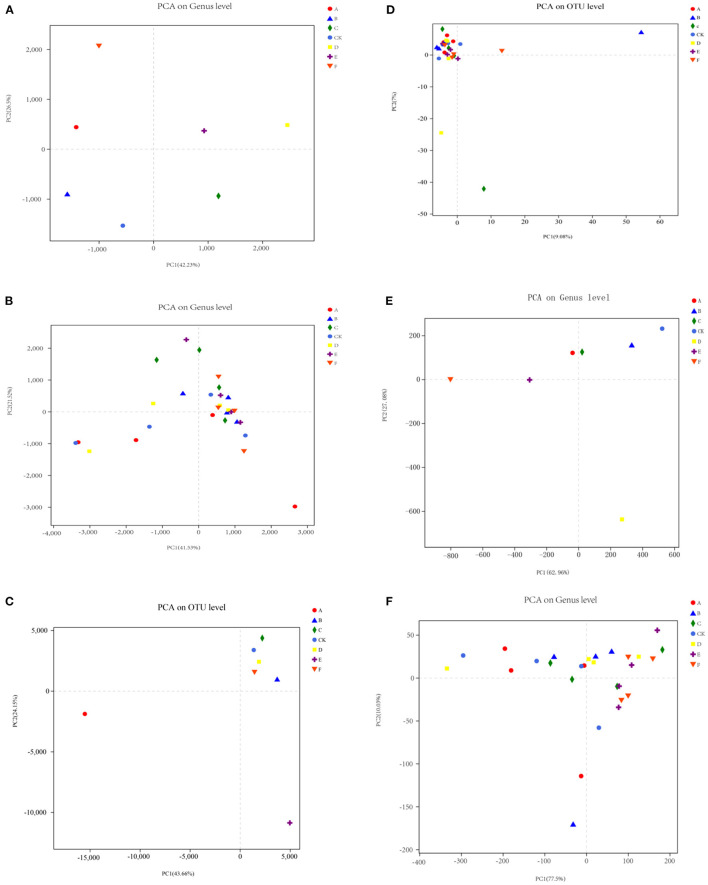
Principal component analysis of the microbial community of the kiwifruit soil after sulfur treatment. **(A,B)** bacteria; **(C,D)** fungi; **(E,F)** actinomycetes.

The main components of the PCA of the soil microbial community were carried out at the genus level. After 1 year of sulfur application, the explanatory degrees of the main component 1 and main component 2 to soil bacterial community composition were 42.23 and 26.50%, respectively. As shown in Figure 4A, in the first main component, different sulfur content had significant differences in the location distribution of bacteria in the rhizosphere soil of kiwifruit. There was a considerable difference in the location distribution of bacteria between sulfur application and no sulfur application in the second main component. It indicated that the sulfur application and its content mainly caused the difference in bacterial community composition in the rhizosphere soil of kiwifruit. After 2 years of sulfur application, the explanatory degrees of main component 1 and main component 2 to soil bacterial community composition were 41.53 and 21.52%, respectively. The bacterial community structure was gradually clustered, and sulfur treatment (S_1.0_, S_1.5_, S_2.0_, S_2.5_, and S_3.0_) could be pressed into one group on the right side of the coordinate axis. S_0.5_ and S_0_ treatments can be grouped and located on the left side of the axis. The results showed that the bacterial community composition of the first main component of kiwifruit was affected by the sulfur application. The low sulfur application had little effect on soil bacterial community composition (Figure 4B).

The explanatory rates of main component 1 and main component 2 in the soil actinomycetes community after 1 year of sulfur application were 62.96 and 27.08%, respectively. The soil actinomycetes community structure was significantly different between the treatments of S_1.0_, S_1.5_, and S_2.0_ and the treatments of S_0.5_, S_2.5_, and S_3.0_ on the right side of the axis. In the first principal component, the sulfur application is the key factor in the difference in kiwi soil's actinomycetes community composition. The second main component showed that the difference in the composition of the actinomycetes community in the rhizosphere soil of kiwifruit is related to the sulfur concentration (Figure 4C). After 2 years of sulfur application, the explanatory degrees of the main component 1 and main component 2 reached 77.5% and 10.03%, respectively. Among all treatments, except the 0.5 kg/m^3^ sulfur treatment, other treatments could be grouped into one group, indicating no significant difference in the composition of the actinomycetes community in the soil of kiwifruit after 2 years (Figure 4D).

After the sulfur application for 1 year, main component 1 and main component 2 accounted for 43.66 and 24.15%, respectively, of the soil fungal community composition. S_0_, S_1.0_, S_1.5_, S_2.0_, and S_3.0_ were grouped into one class on the right side of the coordinate axis. In addition, S_2.5_ and S_0.5_ were grouped into another category. According to the first and second principal components, there were significant differences in the location distribution of fungi, indicating that the content of sulfur application was the main factor leading to the difference in the fungal community structure of kiwifruit (Figure 4E). After 2 years of sulfur application, the explanatory degrees of main component 1 and main component 2 reached 9.08% and 7%, respectively. All treatments in the figure are located on the left side of the coordinate axis and can be grouped into one category, indicating no significant difference in fungal community composition after 2 years of sulfur application (Figure 4F).

## Discussions

Many researchers have found that soil microorganisms profoundly impact the growth, nutrition, and health of plants in agricultural ecosystems (Berendsen et al., 2012; Stephanie, 2018; Islam et al., 2020; Hicks et al., 2021). The quantity of soil microorganisms mainly reflects soil activity and its activity degree (Ma et al., 2017). Microbial biomass carbon content is an important indicator of soil health (Geisseler et al., 2017). Li et al. (2016) studies have shown that sulfur application can significantly increase some soil microbial characteristics. In this study, sulfur application in the range of 1.0~2.0 kg/m^3^ could effectively increase the total microbial biomass of bacteria, actinomycetes, and fungi in the rhizosphere soil of kiwifruit and could effectively improve the soil microbial biomass carbon and nitrogen content. These results indicate that sulfur plays a role in changing the soil microbial structure.

The diversity and uniformity of the soil microbial community structure affected the stability of the ecosystem and improved crops' response-ability to soil microecological environment changes, especially the ability to resist pathogenic microorganisms and crop resistance to disease (Postma et al., 2010; Zhang et al., 2019). Tang et al. (2020) demonstrated that sulfur fertilizers increased the relative abundance of iron (III) and sulfate reduction-related rhizosphere bacterial genera. Confirmed that sulfur could change the diversity of soil microorganisms. By using high-throughput sequencing technology, we investigated the microbial diversity after sulfur application. The results displayed that the Chao index of S_2.5_ treatment was significantly different from that of no sulfur treatment after 1 year of sulfur application. After 2 years of sulfur application, the Shannon, ACE, and Chao indices of kiwifruit soil bacteria increased with increasing sulfur application concentration; however, there was no significant difference in the α diversity of actinomycetes and fungi, indicating that sulfur mainly affected soil bacterial diversity. Further analysis showed that the diversity of each treatment was high at the bacterial genera level after 2 years of sulfur application. With the increase in sulfur application concentration, the proportion of *Acidothermus* in the population increased, which might be due to the oxidation of sulfur to sulfate and the release of H^+^ (Cui et al., 2004). The principal component of bacterial community structure was affected by the amount of sulfur application. In summary, the sulfur application is the key factor leading to microbial differences in kiwifruit rhizosphere soil. Appropriate sulfur could improve microbial structure characteristics of kiwifruit rhizosphere soil, especially the bacterial diversity index and the bacterial community composition structure.

Interestingly, the incidence of kiwifruit canker decreased significantly after sulfur application in the range of 1.0~2.0 kg/m^3^. We believe that this is closely related to the change in soil microbial community after sulfur application. Numerous studies have found that increasing soil microbial diversity can improve crop disease resistance. Soil microorganisms promote crop growth by dissolving nutrients (P, K, and Zn), fixing nitrogen, and chelating iron, as well as by indirectly controlling pathogen growth and alleviating abiotic stress (Kaur et al., 2021). Li et al. discovered that soil microorganisms in a watermelon/wheat companion cropping system can induce resistance of watermelon to *Fusarium wilt* (Li et al., 2019). The application of calcium can increase the resistance of peanuts to soil-borne pathogens by recruiting peanuts with specific dominant bacteria (Zhang et al., 2021). In this study, treatment with sulfur resulted in a significant increase in the proportion of *Acidothermus, norank_f__HSB_OF53-F07*, and *norank_f __Acidobacteriaceae__Subgroup_1*. We speculated that these microbial communities have the potential to improve the resistance of kiwifruit against Psa.

## Conclusion

The results of this study indicated that sulfur supply led to microbial communities' differences in the rhizosphere soil of kiwifruit, significantly increased the bacterial diversity, and changed the composition of the bacterial community. Treatment with sulfur resulted in a significant increase in the proportion of *Acidothermus, norank_f__HSB_OF53-F07*, and *norank_f __Acidobacteriaceae__Subgroup_1*. We speculated that these microbial communities have the potential to improve the resistance of kiwifruit against Psa. The data obtained in this study provide important and novel information about possible alterations in the rhizosphere microbiota in response to sulfur-induced resistance treatment of kiwifruit to bacterial canker.

## Data Availability Statement

The data presented in the study are deposited in the NCBI repository, BioProject ID: PRJNA841390.

## Author Contributions

XY: conceptualization. YL: methodology, validation, and funding acquisition. RS: formal analysis and writing—original draft. SY: resources and data curation. SY and XY: writing—review and editing. JY and SY: investigation. All authors contributed to the article and approved the submitted version.

## Funding

This study was funded by the 13th batch of Guizhou Outstanding Young Scientific and technological Talents Project (Grant No. QKHPTRC5614-2021) and the Ministry of Agriculture and Rural Affairs Science and Technology Education Project, Modern Agricultural Industrial Technology System Post Scientist (Kiwi plant protection technology), 2021–2025.

## Conflict of Interest

The authors declare that the research was conducted in the absence of any commercial or financial relationships that could be construed as a potential conflict of interest.

## Publisher's Note

All claims expressed in this article are solely those of the authors and do not necessarily represent those of their affiliated organizations, or those of the publisher, the editors and the reviewers. Any product that may be evaluated in this article, or claim that may be made by its manufacturer, is not guaranteed or endorsed by the publisher.
